# Persistent dysgeusia post-halitosis treatment: How does it impact the patients’ quality of life?

**DOI:** 10.4317/medoral.22370

**Published:** 2019-05

**Authors:** Bruna-Fernandes-do Carmo Carvalho, Mônica-Ghislaine-Oliveira Alves, Marignês-Theotonio-dos Santos Dutra, Ivan Balducci, Denise Nicodemo, Janete-Dias Almeida

**Affiliations:** 1 Department of Biosciences and Oral Diagnosis, São Paulo State University (Unesp), Institute of Science and Technology, São José dos Campos, São Paulo, Brazil; 2 Brazcubas Educação, Mogi das Cruzes, Brazil; 3 Universidade Mogi das Cruzes, Mogi das Cruzes, Brazil; 4 Department of Social Science and Pediatric Dentistry, São Paulo State University (Unesp), Institute of Science and Technology, São José dos Campos, São Paulo, Brazil

## Abstract

**Background:**

The objective of this study was to evaluate the quality of life and the presence of anxiety and depression in patients with dysgeusia post halitosis treatment.

**Material and Methods:**

Sixty patients were divided into three groups: Dysgeusia group (DG); Halitosis group (HG) and Control group (CG). The following instruments were used: Medical results study Short form health research of 36 items (SF-36), State-state anxiety inventory (STAI) and Self-report questionnaire-20 (SRQ-20).

**Results:**

Of the 60 subjects studied, 85% were female and 15% were male. The evaluation of SF-36 indicated a statistically significant correlation between some domains for DG and showed low scores for Mental Health. In relation to HG, low scores were obtained for Bodily Pain, Vitality and Emotional Role. The CG showed low scores for Bodily Pain, General Health and Vitality. STAI was significant when DG was compared to HG and CG. The mean SRQ-20 score was higher in DG compared with the other groups.

**Conclusions:**

Persistent dysgeusia post-halitosis treatment impacts on QoL generically in the Mental health domain, and specifically in trait and state anxiety. Symptoms of depression were also seen in this group of patients.

** Key words:**Anxiety, depression, dysgeusia, halitosis, oral health, quality of life.

## Introduction

Dysgeusia is a qualitative gustatory disorder characterized by a distortion in the perception of taste. Total dysgeusia is defined as the inability to interpret all basic tastes which, in most cases, is associated with mineral deficiency (1). This condition can cause a loss of appetite and even malnutrition (2).

The inflammation caused by radiotherapy, smoking habit, zinc deficiency, drugs use, medications, chlorhexidine mouthrinse, xerostomia and nerve damage (chorda tympani or glossopharyngeal) can also cause dysgeusia (2,3). In addition, brain tumors, as well as psychiatric, depression and anxiety symptoms, can be associated with alterations in taste (4). Heath *et al.* (5) demonstrated a positive correlation between average trait anxiety scores and bitter and salt taste thresholds.

Dysgeusia is a symptom of difficult measurement, since there is no defined methodology for the determination of its intensity, being therefore 100% based on patient´s complaint. Some studies point out a significant relationship between olfactory and taste disorders (2). The chemosensory cells and taste receptors are not limited to taste buds, as they can be found in airway epithelium, digestive tract, pancreas and brain. Retronasal olfactory function and olfactory nerve endings, transmit nerve impulses to the brain and also helps in the interpretation of the flavors, which reinforce their importance in this process (2).

Halitosis is a disorder characterized by unpleasant odors emanating from the mouth, which can have local causes such as carious processes, presence of a biofilm, gingivitis, fissured tongue, maladjusted dentures, altered salivary composition, tongue coating and periodontal diseases (6,7) or systemic causes such as sinusitis, tonsillitis, rhinitis, bronchitis, abscesses, gastric eruption, duodenal ulcer, diabetes, hormonal alterations and xerostomia (8). The estimated prevalence of halitosis is 50% in the adult population (9). In general, no specific difference in the prevalence or severity of halitosis is observed between genders, but women seem to be more willing to visit health professionals because of this condition (10). Since halitosis is a multifactorial condition, treatment will depend on its origin and can be exclusively local or systemic. In addition, psychological or psychiatric treatment is recommended in cases of pseudohalitosis and halitophobia (11-13).

The microorganisms most commonly causing halitosis are Gram-negative anaerobic bacteria (14), that degrade sulfur-containing amino acids, producing volatile sulfur compounds (VSC) such as hydrogen sulfide (H2S), methyl mercaptan (CH3SH), and dimethyl sulfide (CH3SCH3), which are the main compounds responsible for the foul odor emanating from the mouth. Other compounds such as butyric, propionic and valeric acid and cadaverine are also found, but at lower levels (6,8,10,14).

According to Yaegaki and Coil (12) halitosis has a psychosomatic impact. Eli *et al.* (15) demonstrated that individuals with halitosis can become obsessed with their breath, a fact restricting social behavior and compromising social interactions. Thus, in some patients, even though halitosis is controlled, complaining of bad breath persists, but actually it is related to taste alterations caused by disorders in patient´s gustatory perception or still caused by emotional disturbances (16).

The incidence of halitosis is high in the general population and this condition has a proven psychosomatic impact (11-13,15). However, there are no studies in English literature elucidating the direct correlation between the complaint of dysgeusia post-halitosis treatment. The studies point to dysgeusia as a factor that is often idiopathic or related to zinc deficiency, use of medications and mouthwashes with chlorhexidine (2,3). Once we observed in our daily practice, the trait that some halitosis patients after the treatment exhibited dysgeusia complaint with no obvious cause, we scheduled to study this group separately because we hypothesized that these patients could have psychosomatic alterations with the worst quality of life (QoL). Therefore, the objective of the present study was to evaluate the QoL and presence of anxiety and depression in patients with dysgeusia after halitosis treatment.

## Material and Methods

The present study was approved by the Human Research Ethics Committee of the São Paulo State University (Unesp), Institute of Science and Technology, São José dos Campos (Protocol Nº. 010/2010-PH/CEP). A case-control study was conducted in which the sample consisted of patients seen at Unesp and at a private clinic specialized in halitosis treatment. Patients agreed to participate in the study by signing a free informed consent form.

- Characteristics of the sample 

Sixty patients were randomly assigned into three groups of 20 subjects each as follow:

1- Dysgeusia group (DG): Patients with idiopathic persistent dysgeusia complaint after halitosis’ treatment. In this group, the halitosis was completely resolved, with no further emanation of VSC, confirmed by the portable sulfur monitor (Halimeter®, Interscan, USA). Patients remained with discomfort or insecurity about their breath due to taste alteration. Moreover, patients using medications, presenting periodontal disease with pockets larger than 3 mm, local and systemic causes related to breathe and taste alterations, the presence of Mallampati scores 3 or 4, Brodsky classification grade 3 or 4, and caseous tonsillitis were not included.

2- Halitosis group (HG): Patients diagnosed with halitosis attended in the first appointment, therefore, with untreated halitosis. Patients with a previous diagnosis and treatment for halitosis were not included in this group. The organoleptic measurement was performed according to Grigor *et al.* (17). It was conducted by the same professional previously trained on the ability to detect odors. In addition, VSC produced by the participants were measured by a portable sulphur monitor (Halimeter®, Interscan, USA) and the threshold limit was stated as 150 ppb (17).

3- Control group (CG): Patients without halitosis or dysgeusia complaint.

**Figure 1:**
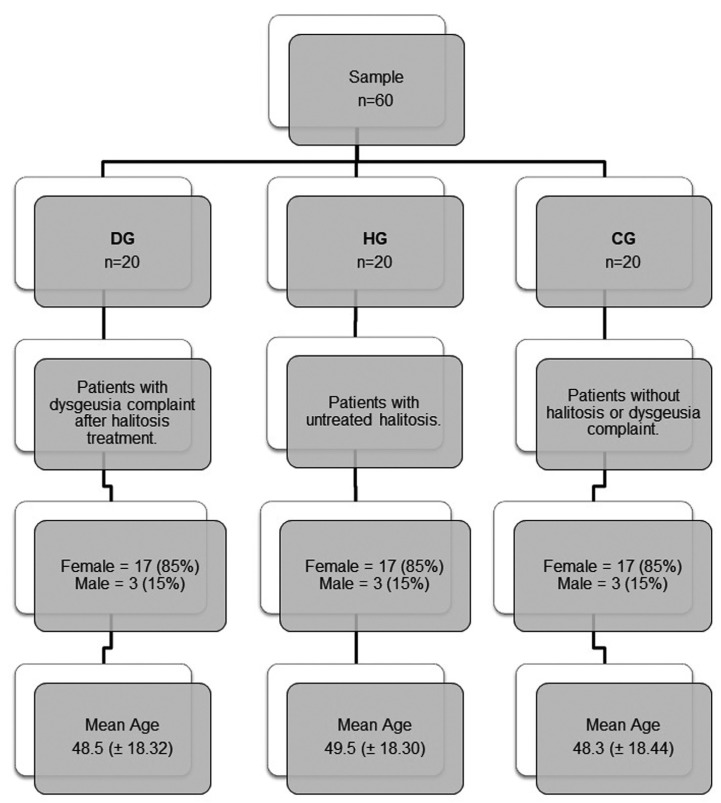
Characteristics of the samples (n=60). Dysgeusia group - DG, Halitosis group - HG, Control group - CG.

Non-inclusion criteria for all groups were the use of illicit drugs, confirmed thyroid disorders, radiotherapy and/or chemotherapy, the presence of autoimmune diseases, syndromes and temporomandibular joint dysfunction. The groups and its characterization are summarized in figure 1.

- Clinical treatment

All subjects were submitted to anamnesis, treatment plan and specific clinical tests for the VSC-producing bacteria and diagnosis of halitosis.

The DG received a halitosis standard treatment before the first appointment. This treatment consisted of dental prophylaxis and oral hygiene orientation, such as the use of a specific tongue cleaner scraper and a tongue cleaner (Cetylpyridinium Chloride spray). The patients were also instructed to clean the nasal fossae and throat using warm saline irrigation when mucus was present in the nose or oropharynx and in the case of flu, colds and/or rhinitis. They also received instructions to prevent prolonged fasting (hypoglycemia); to control salivary flow through mechanical and gustatory stimulants of salivary secretion; to control water intake; to control the consumption of foods with high animal protein content. Post-halitosis’ treatment, the patients presented a complaint of persistent dysgeusia and had performed vitamin deficiency or insufficiency analysis. In patients with low serum zinc levels, supplementation was administered because it is related to the general gustatory function that can affect the general mood scores in patients with dysgeusia (18).

The HG’s patients received the same standard treatment for halitosis as described in DG. Since HG patients did not present dysgeusia complaint, no vitamin deficiency investigation or any supplementation was done.

- QoL Questionnaires 

**Figure 2:**
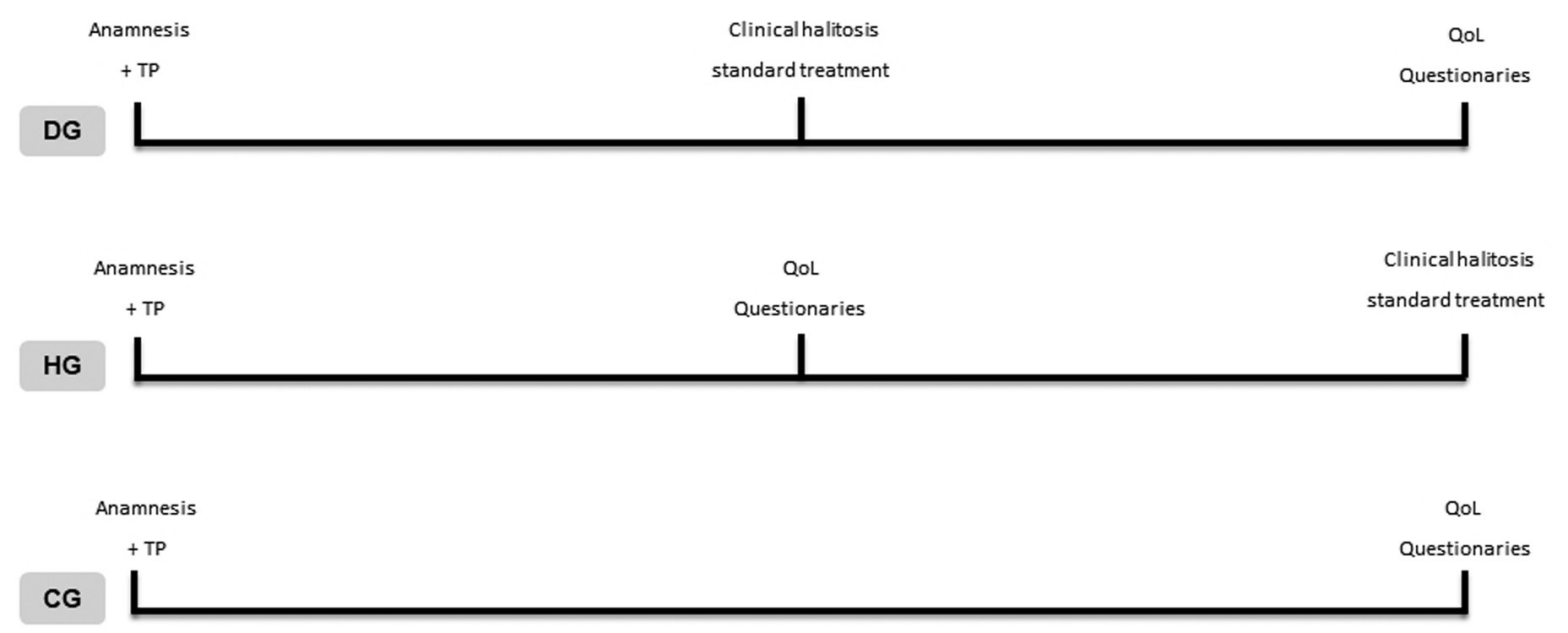
The experimental design. Dysgeusia group (DG), Halitosis group (HG), Control group (CG), Treatment plan (TP), Quality of life (QoL).

The emotional profile of the subjects studied was evaluated using instruments for assessment of generic and specific aspects of health-related QoL. The QoL questionnaires were applied as follows: in the DG, the patients answered the questionnaires after the halitosis treatment. In the HG, the patients answered the questionnaires before the clinical halitosis standard treatment. In the CG the questionnaires were applied at the first appointment, after the anamnesis. The experimental design is shown in figure 2.

For all instruments, the patients only marked the answers that were closest to what they considered to be true. The patients completed the self-administered questionnaires in a quiet room after instruction by the examiner. The following instruments were used: Medical Outcomes Study 36-Item Short Form Health Survey (SF-36), State-Trait Anxiety Inventory (STAI), and Self-Reporting Questionnaire-20 (SRQ-20).

The SF-36 is a generic questionnaire used to assess QoL, which emphasizes the individual’s perception of health in the last 4 weeks and consists of eight domains divided into two main groups: physical (physical functioning - PF, role physical - RP, bodily pain - BP, general health - GH) and mental (vitality - V, social functioning - SF, role emotional - RE, and mental health - MH). The scores obtained for each domain range from 0 (poor health) to 100 (good health).

The STAI is a self-report questionnaire designed to measure anxiety, which depends on the conscious reflection of the subject in evaluating his state of anxiety, as well as characteristics of his personality. The questionnaire is designed to measure state anxiety (transient emotional state) and trait anxiety (the tendency of how to respond to situations). The STAI consists of two 20-item scales. In the trait anxiety scale (STAI-T), the subjects are asked to indicate how they generally feel, and in the state anxiety scale (STAI-S) how they feel at this very moment. Scores of 20 to 34 indicate low anxiety, 35 to 49 moderate anxiety, 50 to 64 high anxiety, and 65 to 80 very high anxiety.

The SRQ-20 was designed to screen for psycho-emotional disorders at primary care services with the objective to detect non-psychotic diseases. The questionnaire consists of 20 questions to be answered yes (1) or no (0). The final score is given by the sum of yes answers, with a score of 8 or higher indicating a state of depression.

- Statistical analysis

Descriptive analysis of the results consisted of measures of central tendency (mean) and dispersion (standard deviation) calculation. For inferential analysis, one-factor analysis of variance by the Holm-Sidak test and Pearson’s linear correlation coefficient were used. A level of significance of 5% was adopted.

## Results

Of the 60 subjects studied, groups were matched for gender and age (± 3 years) at a proportion of 1:1 to DG and the variables are shown in Figure 1.

The groups in study were compared at each domain of SF-36. The results of these comparisons are shown in  Table 1. The domains with the lowest scores were vitality, role emotional and mental health in DG; bodily pain, vitality and role emotional in HG, and bodily pain, general health and vitality in CG. For MH domain, ANOVA and Sidak’s multiple comparisons test (5%) indicated that DG differed from CG.

**Table 1:**
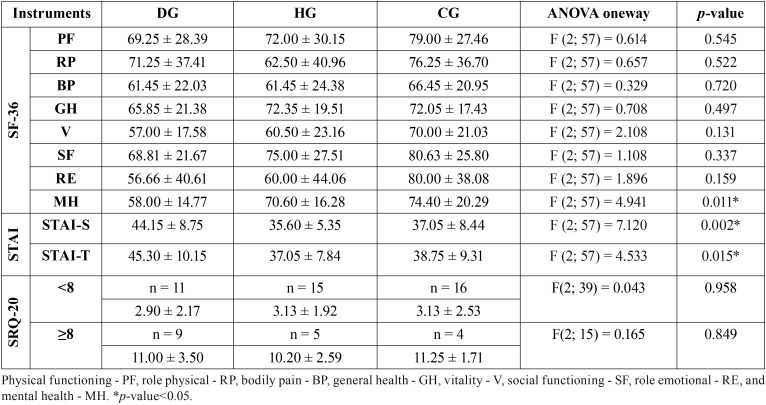
Comparison of groups: Descriptive statistics and results of ANOVA oneway for SF-36, STAI and SRQ-20.

A correlation analysis between the eight domains of SF-36 was made. For DG patients the following domains were statistically significant correlated: (i) PF versus BP; PF versus GH; (ii) RP versus BP; (iii) RE versus BP and (iv) MH versus GH. The correlations coefficients of Pearson obtained in these cases were positive and moderated.

The groups were compared for STAI instrument. The higher scores indicate a high level of anxiety. Higher scores to STAI-S and STAI-T were observed in DG when compared to HG and CG. The ANOVA oneway test was applied to STAI-S and STAI-T. A significant statistical difference was observed in STAI-S means between DG and the other groups (HG and CG); while for STAI-T, a significant statistical difference was observed between DG and HG ( Table 1).

Depressive symptoms (scored SRQ-20 ≥ 8) were present in 45% (9/20) in DG while, in HG in 25% (5/20). However, the highest mean score was observed in DG (6.55 ± 4.97). The lowest mean score mean scores was observed in CG (4.75 ± 4.08). A comparison between the mean values of the three groups was performed by the Holm-Sidak test (5%) that revealed no significant difference between the mean scores ( Table 1).

Correlation analysis between the variables STAI and SRQ-20 was made for better understanding the behavior of patients with anxiety and depression. Pearson’s correlation between the STAI-S, STAI-T and SRQ-20 in DG showed a significant moderate positive correlation between the SRQ-20 and STAI-S (r = 0.561 and *p* = 0.010 < 0.05) and between the SRQ-20 and STAI-T (r = 0.687 and *p* = 0.001 < 0.05), as well as a significant strong positive correlation between the STAI-S and STAI-T (r = 0.783 and *p* = 0.001 < 0.05). In HG, there was a significant moderate positive correlation between the STAI-S and STAI-T (r = 0.503 and *p* = 0.024 < 0.05) and a significant strong positive correlation between the SRQ-20 and STAI-T (r = 0.821 and *p* = 0.001 < 0.05). In CG, a significant moderate positive correlation was observed between the SRQ-20 and STAI-T (r = 0.660 and *p* = 0.002 < 0.05) and between the STAI-S and STAI-T (r = 0.698 and *p* = 0.001< 0.05), and a significant strong positive correlation between the SRQ-20 and STAI-S (r = 0.719 and *p* = 0.001 < 0.05).

## Discussion

Dysgeusia was observed as a complaint in patients post halitosis treatment. To the best of our knowledge, this is the first study to describe this aspect and to evaluate the QoL in this specific group of patients. This study was important to understand the impact of dysgeusia on patients’ QoL.

Studies investigating psychosomatic disorders in patients with dysgeusia are sparse in the international literature. In addition, many dental professionals are unaware of the importance of their role in the treatment of manifestations of dysgeusia and halitosis.

Dysgeusia is defined as an altered sense or loss of taste, in which the perception of sour and bitter is affected first, followed by the perception of sweet and salty (5). Organic alterations such as atrophy of the tongue papillae are observed in cases of dysgeusia with mineral deficiency, particularly zinc deficiency (19). In contrast, most cases of halitosis originate in the oral cavity from microbial putrefaction or bacterial metabolism of amino acids in local tissue debris, generating the strong odor characteristic of halitosis (6,14,23).

However, we point out that the exclusion criteria were not common to the groups as each group has specific criteria. For the DG, it was necessary to exclude the use of drugs, because the literature indicates that some drugs may promote changes in the taste buds (2). Also, the presence of local and systemic causes related to respiratory and gustatory alterations was excluded, since the patients had already undergone halitosis treatment. In GH, there were no exclusion criteria since patients were beginning the halitosis treatment.

In agreement with other reports on halitosis (10,20,21) and dysgeusia (22), higher prevalence of dysgeusia was observed in women in the present study. This finding suggests that women are more willing to seek a professional for resolution of these oral problems. The mean age of the present patients with dysgeusia differs from Doty *et al.* (22) who found a lower mean age for both genders. The mean age of patients with halitosis was 49.5 years. Mean ages of 35.12 years (21), 43.7 years (23) and 51.9 years (20) have been reported in other studies.

When analyzing the domains of the SF-36, which is a generic instrument for the assessment of QoL, it should be remembered that each domain evaluates a different aspect of the individual (24): physical functioning domain evaluates how an individual performs daily tasks; the role physical domain evaluates how physical health interferes with habitual domestic or professional activities; the bodily pain domain indicates how much pain the individual experienced in the last weeks and the limitations imposed by it in daily living; the general health domain evaluates the individual’s perception of his own health and his expectations for the future; the vitality domain indicates the level of energy and disposition of the individual to perform daily tasks; the social functioning domain evaluates how much an individual’s habitual social activities are affected by his physical or emotional state; the role emotional domain evaluates how the emotional state interferes with daily domestic or professional activities, and the mental health domain evaluates how much of the time the individual feels anxious and depressed or happy and calm in daily living.

Mean SF-36 scores results for the mental health domain were lower in DG, indicating that the emotional state of these patients interferes negatively with their daily activities, a fact not observed for CG. A positive correlation was observed between mental health and general health status in DG, a finding highlighting how much these patients feel anxious and depressed and demonstrating the negative interference with the perception of their own health. Boltong *et al.* (25) indicated the need for a better understanding of the problems related to altered taste and their impact on QoL, as well as the need for adequate assessment tools.

The STAI has been validated for the Portuguese language (26) and is one of the most commonly used instruments to quantify subjective components related to anxiety (27). The objective of the STAI-T is to evaluate how an individual copes with anxiety throughout life, whereas the STAI-S refers to the response to anxiety at the time of assessment (26).

Part of the patients of DG (n = 9, 45%) presented significantly higher levels of anxiety in both the STAI-S and STAI-T. In agreement with the study of Heath *et al.* (5), a positive correlation was observed between personality trait (STAI-T) and depressive symptoms (SRQ-20) in DG. Authors also suggested that altered taste in emotional disorders may reflect an actual change in the gustatory system.

Anxiety is a factor frequently associated with cases of halitosis. Settineri *et al.* (21) showed that patients with halitosis have higher trait and state anxiety scores and also report a higher frequency of stress-related problems. Zaitsu *et al.* (20) observed that patients with genuine halitosis, in whom malodor is caused by processes of putrefaction in the oral cavity, have a strong trait of social anxiety disorders and experience difficulties in overcoming their anxiety about bad breath. This finding might be explained by the fact that halitosis has a negative and direct influence on interpersonal relationships.

In the study of Miller and Naylor (28), taste alterations were reported by patients with disorders linked to depression. Nalcaci and Baran (29) indicated dysgeusia to be a symptom of major depression. These studies suggest that taste alterations are secondary to treatment of emotional disorders and not the other way around. In the present study, the number of patients with high SRQ-20 scores, i.e., subjects with depressive symptoms, was higher in DG. In general, patients with dysgeusia scored higher in this questionnaire than those of the other groups.

Dysgeusia is an atypical and neglected symptom of depression and little is known about the mechanisms whereby anxiety and depression disorders cause taste disturbance (5). Tanaka *et al.* (19) reported the presence of psychological alterations in 24 (55.8%) of 43 patients with dysgeusia and in 48 (90.6%) of 53 patients with glossodynia. Taste is a key sense in the regulation of essential nourishment and for overall health. Any disturbance in taste perception can influence appetite, body weight and psychological well-being, impacting the QoL of these patients (2).

Pseudohalitosis has been observed to be associated with the worst quality of life (11-13,30). Although the present study did not establish a direct relation between, we suggest that pseudohalitosis and dysgeusia may be associated since patients with persistent dysgeusia had halitophobia even without VSC. Thus, other studies are necessary to better investigate the relationship between these complaints.

Oral sensorial complaints comprise a broad and complex theme. It has a broad possibility of discussion, and yet, no gold standard treatment is available. It is a really promising field of research. In the present study, authors concluded that persistent dysgeusia post-halitosis treatment impacts on QoL generically in the Mental health domains, and specifically in trait and state anxiety. Symptoms of depression were also seen in this group of patients.
